# Pannexin1 Is Associated with Enhanced Epithelial-To-Mesenchymal Transition in Human Patient Breast Cancer Tissues and in Breast Cancer Cell Lines

**DOI:** 10.3390/cancers11121967

**Published:** 2019-12-07

**Authors:** Nour Jalaleddine, Layal El-Hajjar, Hassan Dakik, Abdullah Shaito, Jessica Saliba, Rémi Safi, Kazem Zibara, Marwan El-Sabban

**Affiliations:** 1 Department of Biological and Environmental Sciences, Faculty of Science, Beirut Arab University, Beirut 1107-2809, Lebanon; nourjalaleddine@hotmail.com; 2 Department of Anatomy, Cell Biology and Physiological Sciences, Faculty of Medicine, American University of Beirut, Beirut 1107-2020, Lebanon; lh85@aub.edu.lb; 3 University of Tours, EA 7501 GICC, CNRS ERL 7001 LNOx, CEDEX 01, 37032 Tours, France; hassan.da89@live.com; 4 Department of Biological and Chemical Sciences, Faculty of Arts and Sciences, Lebanese International University, Beirut 1105, Lebanon; abdshaito@gmail.com; 5 Department of Biology, Faculty of Sciences, Lebanese University, Hadath, Beirut 1003, Lebanon; jessicasaliba@hotmail.com; 6 Department of Dermatology, Faculty of Medicine, American University of Beirut, Beirut 1107-2020, Lebanon; rs176@aub.edu.lb; 7 ER045-Laboratory of Stem Cells, PRASE, Department of Biology, Faculty of Sciences, Lebanese University, Hadath, Beirut 1003, Lebanon; kzibara@ul.edu.lb

**Keywords:** cellular communication, Pannexin1, epithelial-to-mesenchymal transition, gene set enrichment analysis (GSEA), metastasis

## Abstract

Loss of connexin-mediated cell-cell communication is a hallmark of breast cancer progression. Pannexin1 (PANX1), a glycoprotein that shares structural and functional features with connexins and engages in cell communication with its environment, is highly expressed in breast cancer metastatic foci; however, PANX1 contribution to metastatic progression is still obscure. Here we report elevated expression of PANX1 in different breast cancer (BRCA) subtypes using RNA-seq data from The Cancer Genome Atlas (TCGA). The elevated PANX1 expression correlated with poorer outcomes in TCGA BRCA patients. In addition, gene set enrichment analysis (GSEA) revealed that epithelial-to-mesenchymal transition (EMT) pathway genes correlated positively with PANX1 expression. Pharmacological inhibition of PANX1, in MDA-MB-231 and MCF-7 breast cancer cells, or genetic ablation of PANX1, in MDA-MB-231 cells, reverted the EMT phenotype, as evidenced by decreased expression of EMT markers. In addition, PANX1 inhibition or genetic ablation decreased the invasiveness of MDA-MB-231 cells. Our results suggest PANX1 overexpression in breast cancer is associated with a shift towards an EMT phenotype, in silico and in vitro, attributing to it a tumor-promoting effect, with poorer clinical outcomes in breast cancer patients. This association offers a novel target for breast cancer therapy.

## 1. Introduction

Breast cancer remains the primary cause of mortality in women worldwide [1]. Breast cancer is a heterogeneous disease showing high molecular variability. Whole-genome gene expression profiling based on microarray comparisons has classified breast cancer into subtypes according to their molecular signatures [2,3]. These microarray signatures lead to the stratification of breast cancer patients based on their level of expression of estrogen receptor alpha (ERα), progesterone receptor (PR), or the human epidermal growth factor receptor 2; ERBB2 (HER2) [2,3]. Molecular profiling classified breast cancer into the following “intrinsic subtypes of breast cancer”: Normal-like, Luminal A (ER^+^ PR^+/−^ HER2^−^), Luminal B (ER^+^ PR^+/−^ HER2^+^ and/or Ki67^+^), HER2-enriched (PR^+^, and over-expression of HER2 due to a genomic amplification ERBB2) with around half of HER2-enriched tumors expressing ERα, and basal-like (ER^−^ PR^−^ HER2^−^ and expressing basal cytokeratins, triple negative, TNBC) [2,3,4,5]. Further molecular profiling is expected to stratify the known intrinsic breast cancer subtypes into new subclasses, for example, the recently identified claudin-low basal-like breast cancer [4,6]. Most importantly, the intrinsic cancer subtypes show clinical heterogeneity, as well, being different in incidence, patient survival, and response to treatment [6].

Metastasis is a multistep process during which cancer cells spread from the tumor of origin to colonize distant sites, a process indicative of cancer progression. Different cancers preferentially metastasize to specific organs [7,8,9]. In breast cancer, metastasis is mostly directed to lungs, bones, and brain [10,11]. Metastasis involves intrinsic (genetic) and extrinsic (non-genetic) factors [12,13] and accounts for over 90% of cancer mortality and morbidity [14]. Intercellular communication and communication between the cell and its extracellular environment play a critical role in maintaining homeostasis [15,16] and are involved in cancer progression and the dissemination of cancer cells [12]. In this context, metastasis is largely attributed to the disruption of communication between cells and their extracellular microenvironment [12].

Pannexins (PANX) are vertebrate membrane-spanning proteins [17] that mediate communication between cells and the extracellular environment [18,19,20,21]. PANXs share similar topology with gap junction-forming proteins, connexins [22,23], yet, their ability to form cell-cell junctions remains controversial [23,24]. In humans, 3 PANXs, namely, PANX1, PANX2, and PANX3, have been described. PANX1 is the best characterized pannexin, being implicated in several human diseases [17,18,25,26]. Little is known about its role during tumorigenesis or its mechanistic involvement in breast cancer progression [27,28,29]. Furlow et al. have identified PANX1^1–89^, a C-terminal domain truncated form of PANX1, as a highly expressed protein in metastatic breast cancer cells, and as a pro-metastatic marker promoting cancer cell survival in the vasculature [30]. Moreover, Stewart et al. have studied the role of PANX1 during normal mammary gland development and in silico in human breast cancer tissues. They described elevated PANX1 expression in alveolar development following mammary gland transition into pregnancy and early lactation in mice. They also reported increased PANX1 expression as breast cancer progressed in human patients. Notably, PANX1 overexpression was correlated with poor clinical outcomes in terms of overall survival and progression-free survival [31]. In contrast, others have reported that PANX1 could be a tumor suppressor. For instance, PANX1 was reported to play tumor suppressor roles in C6 glioma cells [29], in squamous and basal cell carcinomas of the skin [32], and in rhabdomyosarcoma [33]. This controversy in the role of PANX1 during cancer progression could be due to differences in the type or stage of cancer [31].

Epithelial-to-mesenchymal transition (EMT) [34] is a hallmark of carcinogenesis, being involved in cancer progression as well as metastasis by enhancing the loss of contact inhibition, stimulating cell motility, and promoting cell invasiveness [35,36,37]. Epithelial-to-mesenchymal transition is characterized by the loss of epithelial phenotypes and the acquisition of mesenchymal characteristics [35,36,37,38,39], including the disruption of cell-cell junctions, reorganization of the actin cytoskeleton, and increased expression of mesenchymal genes and reduced expression of epithelial genes [40]. An enhanced EMT profile and increased expression of mesenchymal markers such as N-cadherin and vimentin are correlated with increased cell invasiveness [36]. EMT involvement in breast cancer has been shown in vitro, in normal and malignant mammary epithelial cells, and in vivo, in mice [41]. Breast cancer stem cell generation and maintenance require EMT signaling [42]. During cancer progression, the induction of EMT engages a complex network of signaling molecules involving TGF-β, Wnt, Notch, NFκB, and ERK/MAPK pathways [43]. In cells undergoing EMT, nuclear localization of β-catenin activates target genes including EMT genes [41,44]. The β-catenin/TCF/LEF protein complex directly upregulates genes associated with EMT, such as the EMT transcription factors Snail1 and Zeb1 [41,44,45]. Snail1 and Zeb1 act as transcriptional repressors of E-cadherin, leading to down-regulation of E-cadherin expression during EMT [46,47].

We have evaluated the contribution of PANX1 expression in a collection of breast cancer (BRCA) RNA-seq dataset available in The Cancer Genome Atlas (TCGA) database and correlated PANX1 expression with overall survival (OS). In addition, gene set enrichment analysis (GSEA) was performed to find pathways enriched in breast cancer, based on PANX1 expression. To support in silico data, breast cancer cell lines MDA-MB-231 and MCF-7 were used. Pharmacological inhibition by probenecid [48] or CRISPR/Cas9 gene knock-out of PANX1 channels were performed and their effects on EMT regulation were evaluated in both cell lines. 

This report provides evidence that upregulated PANX1 expression, in different breast cancer subtypes, enhances EMT phenotype in silico and in vitro and is associated with poorer breast cancer prognosis.

## 2. Results

### 2.1. PANX1 Over-Expression Is Correlated With Poorer OS in Breast Cancer Patients

**Figure 1:**
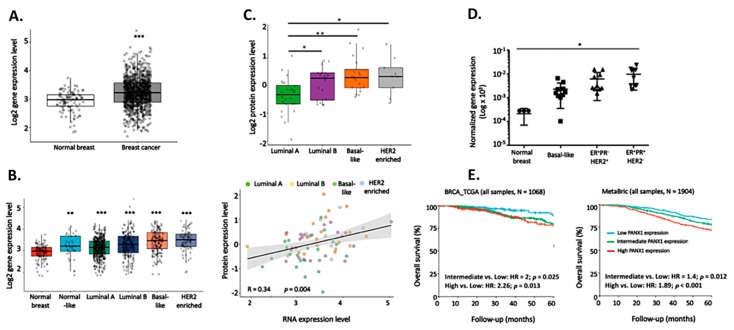
PANX1 over-expression is correlated with poorer overall survival (OS) in breast cancer patients. (**A**) RNAseq data of 1170 primary breast cancer tissues and 109 adjacent normal breast tissue samples were obtained from The Cancer Genome Atlas (TCGA) breast cancer (BRCA) dataset. Gene expression values were represented as Fragment Per Kilobase Million mapped reads (FPKM). Prior to analysis, FPKM values were transformed to log2 scale. PANX1 mRNA levels were compared between the normal and BRCA samples. (**B**) Increased PANX1 expression in all breast cancer subtypes compared to normal breast tissues. The 1071 TCGA BRCA samples were subdivided into the intrinsic BRCA subtypes, and then their PANX1 RNA expression was compared to PANX1 mRNA levels in normal breast cancer tissues. (**C**) Upper panel: Proteomics analysis of PANX1 protein levels in the intrinsic breast cancer subtypes. iTRAQ proteomics data of 72 TCGA BRCA samples were collected from the Clinical Proteomic Tumor Analysis Consortium (CPTAC) data portal. Protein expression was calculated as log2 Ratio to a pooled internal reference comprising a mixture of 40 samples with equal representation of the 4 breast cancer subtypes. Lower panel: Scatter plot showing the correlation between PANX1 protein and mRNA levels in the 72 samples of intrinsic breast cancer subtypes. Protein expression is presented as log2 Ratio while mRNA abundance is presented as log2 expression level. Linear regression alongside is 95% confidence interval (gray area). Pearson’s correlation coefficient (R) and its p-value are denoted in the bottom-left corner. (**D**) qRT-PCR of PANX1 mRNA levels in a cohort of breast carcinoma patients of different subtypes: TNBC tissues *N* = 11; ER^+^ PR^−^ HER2^+^ *N* = 11; ER^+^ PR^+^ HER2^−^ *N* = 15. Patients were females with no prior therapy, selected according to the immune-histochemical tumor expression profile of ER, PR, and HER2. Normal breast tissues were obtained from breast tissue of patients who underwent reduction mammoplasty. (**E**) OS Kaplan Meier plots of the BRCA TCGA (left) and the Molecular Taxonomy of Breast Cancer International Consortium (METABRIC, right) breast cancer patients. The TCGA (*N* = 1068) and METABRIC (*N* = 1904) BRCA samples were divided into Low, Intermediate, or High PANX1 expression groups based on the 25th and 75th percentiles of PANX1 expression. Kaplan Meier plots were used to compare OS of High/Intermediate versus Low PANX1 expression groups. * *p* < 0.05, ** *p* < 0.01, and *** *p* < 0.001.

To investigate the role that PANX1 plays during cancer progression, we compared PANX1 expression between primary breast carcinoma (BRCA) using the RNA-seq dataset obtained from The Cancer Genome Atlas (TCGA). The TCGA data set contained 1180 breast tissue samples in total; 1170 breast cancer samples and 109 samples from breast tissue adjacent to the primary tumor that were considered normal in this study. PANX1 mRNA levels are significantly higher in breast cancer patient tissues than in the normal non-cancerous adjacent tissues (Figure 1A).

Significantly higher PANX1 mRNA levels were seen in all of the intrinsic breast cancer subtypes when compared to normal breast cancer tissues of the TCGA data set (Figure 1B). Compared to Luminal A (ER^+^ PR^+^ HER2^−^) breast cancer subtype, Luminal B (ER^+^ PR^+^ HER2^+^), TNBC and HER2-enriched subtypes showed significantly higher expression of PANX1. In fact, PANX1 was elevated in the different breast cancer subtypes not only at the transcriptional levels but also at the protein levels, as determined by Proteomics analysis of PANX1 protein levels in the intrinsic breast cancer subtypes (Figure 1C). At the protein level, PANX1 had higher levels in HER2-enriched, TNBC, and Luminal B compared to Luminal A, which had the lowest PANX1 protein levels (*p* < 0.05 and *p* < 0.01) (Figure 1C, upper panel). In addition, the levels of PANX1 protein and mRNA were correlated in the different intrinsic breast cancer subtypes (R = 0.34, *p* = 0.004) (Figure 1C, lower panel).

Using qRT-PCR, we also investigated the expression of PANX1 in primary breast cancer tissues from a local cohort of archived breast cancer patients’ samples. PANX1 mRNA levels were up-regulated in basal-like TNBC tissues (*N* = 11) and in HER2^−^ (*N* = 15) and HER2^+^ (*N* = 11) breast cancer subtypes, as compared to normal breast tissue obtained from subjects who underwent reduction mammoplasty; though statistical significance was only reached in the HER2^–^ subtype with *p* < 0.05 (Figure 1D). These data indicate that PANX1 is upregulated, yet differentially in the different subtypes of breast cancer.

The elevated PANX1 expression in TCGA breast cancer tissues is correlated with clinical outcomes. In the TCGA dataset, BRCA patients with high or intermediate PANX1 expression had worse overall survival (OS) compared to patients with low expression (intermediate vs. low: HR = 2, *p* = 0.025; High vs. Low: HR = 2.26, *p* = 0.013) (Figure 1E, left panel). Remarkably, PANX1 was of prognostic value in a microarray dataset from the Molecular Taxonomy of Breast Cancer International Consortium (METABRIC) (intermediate vs. low: HR = 1.4, *p* = 0.012; high vs. low: HR = 1.89, *p* < 0.001) (Figure 1E, right panel). Analysis showed that PANX1 gene expression levels were not age-dependent in breast cancer tissue (*p* = 0.904, Figure S1) or in adjacent non-cancer breast tissue (*p* = 0.892, Figure S1).

### 2.2. EMT Pathway Correlates Positively with PANX1 Expression

**Figure 2:**
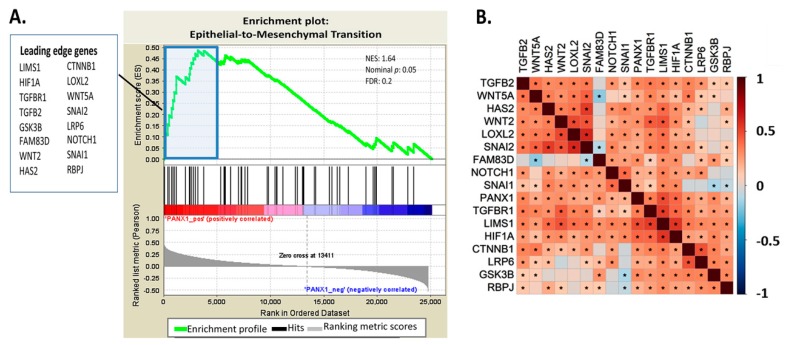
EMT pathway gene expression correlates positively with PANX1 expression. (**A**) Gene set enrichment analysis (GSEA) comparing Low versus High PANX1 expression in the TCGA BRCA patients was run on gene ontology (GO) database. Differential gene expression was ranked by fold change using the Pearson correlation method. Genes that are most positively correlated with PANX1 are shown on the left (red zone) while those that are negatively correlated are shown on the right (blue zone). The false discovery rate (FDR) was calculated by doing 1000 phenotype permutations. The 16 EMT genes from the leading edge are highlighted. (**B**) Correlation plot showing the correlation between PANX1 expression and the 16 EMT genes found in the leading edge. Heatmap colors reflect Pearson correlation coefficients with red for positive correlation and blue for negative correlation. An asterisk indicates correlation significance with BH-adjusted.

To gain a mechanistic insight into the effect of PANX1 overexpression in BRCA tissues, GSEA based on PANX1 expression in BRCA patients was run on the KEGG database and the gene ontology (GO) database. Three cell adhesion-related pathways, including adhaerens junction, focal adhesion, and gap junctions gene set, were among the highly enriched pathways in the KEGG database analysis (data not shown). GSEA analysis of the GO database revealed that the EMT pathway was one of the top enriched GO pathways, based on PANX1 expression (Figure 2A). Figure 2A also shows 16 highly enriched EMT genes that form the leading edge of the enrichment plot. In addition to their high correlation with PANX1 expression, the 16 EMT genes of the leading edge are also highly inter-correlated (Figure 2B). Therefore, PANX1 upregulation in the BRCA TCGA clinical samples correlated with alterations in the EMT pathway genes.

Notably, β-catenin (CTNNB1), known to regulate the EMT pathway, is among the genes highly correlated with PANX1 upregulation (Figure 2). This result shows that PANX1 overexpression correlates with upregulation of β-catenin expression.

### 2.3. PANX1 Channel Permeability Inhibition Reduces Cell Viability and Induces Cell Cycle Arrest in Breast Cancer Cell Lines

**Figure 3:**
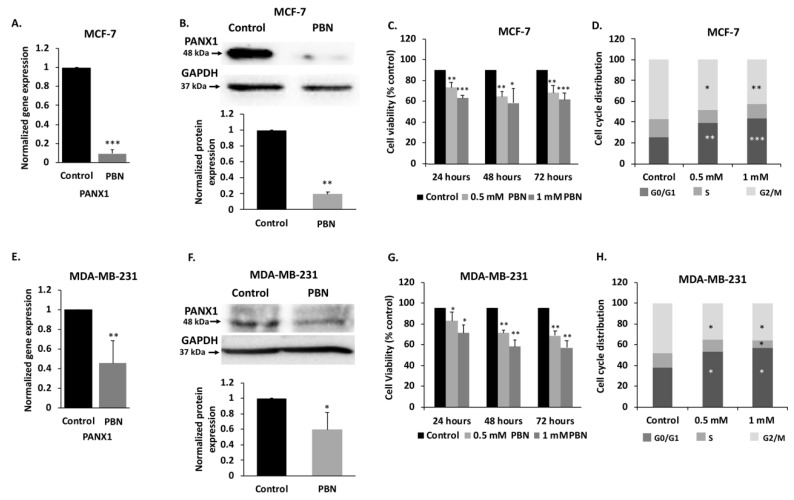
PANX1 channel permeability inhibition reduces cell viability and induces cell cycle arrest in MDA-MB-231 and MCF-7 breast cancer cell lines. (**A**) mRNA levels of PANX1 in MCF-7 cells. qRT-PCR of PANX1, normalized to glyceraldehyde-3-phosphate dehydrogenase GAPDH, in cells treated with 1 mM PBN for 72 h. (**B**) Analysis of PANX1 protein levels using Western blotting in MCF-7 cells treated with 1 mM PBN for 72 h. Lower panel is a densitometry quantification of the PANX1 and GAPDH bands using Image Lab software. Values represent the average fold change in PANX1 expression, normalized to GAPDH, and relative to control, for a total of three Western blots. (**C**) Cell viability of MCF-7 cells treated with PBN was assessed by trypan blue dye exclusion assay. Cells were treated with 0.5 or 1 mM PBN for 24, 48 or 72 h. Average cell viability of three independent experiments is displayed as percentage of control (Details of whole blot can be found at Figure S3 and Table S1). (**D**) Cell cycle distribution analysis was performed by flow cytometry for MCF-7 cells treated with 0.5 mM and 1 mM PBN for 72 h and stained with propidium iodide for cell cycle analysis. Histogram displays averages from three independent experiments. (**E**,**F**) Same as panels A and B, but for MDA-MB-231 cells treated with 1 mM PBN for 72 h. (**G**) Same as (C) but for MDA-MB-231 cells treated with 0.5 or 1 mM PBN for 24, 48 or 72 h. (**H**) Same as in (D) but MDA-MB-231 cells were used instead. Control denotes DMSO vehicle treated cells. * *p* < 0.05, ** *p* < 0.01 and *** *p* < 0.001.

In order to validate the in silico data, we conducted in vitro assays, using luminal type MCF-7 and the highly invasive TNBC MDA-MB-231 cell lines. qRT-PCR and Western blotting analysis showed that PANX1 mRNA and protein are expressed in both MCF-7 (Figure 3A,B) and MDA-MB-231 cell lines (Figure 3E,F).

To assess the effect of PANX1 activity inhibition on PANX1 levels in MCF-7 and MDA-MB-231 cells, cells were treated with probenecid (PBN), a PANX1 channel inhibitor. Probenecid treatment of MCF-7 cells caused a significant decrease in the mRNA (Figure 3A, *p* < 0.001) and protein levels (Figure 3B, *p* < 0.01) of PANX1. Similar trends were observed in MDA-MB-231 cells (Figure 3E,F, *p* < 0.05 and *p* < 0.01).

In addition, PANX1 channel blockade by PBN induced a significant dose-dependent decrease in cell proliferation, in both cell lines, as compared to vehicle-treated control cells (Figure 3C,G), as early as 24 h following PBN treatment in MCF-7 and MDA-MB-231 cells. The reduction in proliferation reached a significant 20% decrease at 48 and 72 h following treatment with 0.5 mM PBN (*P* < 0.01) in both cell lines. A more pronounced decrease of 40% in cell proliferation was detected at 48 and 72 h following treatment with 1 mM PBN, as compared to control cells in both cell lines (Figure 3C, *p* < 0.001 and Figure 3G, *p* < 0.01).

In order to evaluate how PBN inhibits breast cancer cell proliferation, cell cycle analysis was performed at 72 h following PBN treatment. Cell cycle distribution of MCF-7 and MDA-MB-231 cells showed that 0.5 or 1 mM PBN treatment induced cell cycle arrest in the G0/G1 phase (Figure 3D,H), preventing cells from progressing towards mitosis. In fact, a higher percentage of cells were blocked in the G0/G1 phase following 72 h of PBN treatment; MCF-7 cells—39% of cells were arrested at G0/G1 when treated with 0.5 mM PBN (*p*  <  0.01) and 41% when treated with 1 mM PBN (*p*  <  0.001) versus 26% of control cells (Figure 3D); MDA-MB-231 cells—53% of cells were arrested at G0/G1 when treated with 0.5 mM PBN (*p*  <  0.05) and 57% when treated with 1 mM PBN (*p*  <  0.05) versus 38% in control cells (Figure 3H); in addition to a reduced percentage of cells in the G2/M phase in PBN-treated cells compared to control.

These data indicate that PBN has decreased cell growth and viability of both MCF-7 and MDA-MB-231 cells by inducing a cell cycle arrest at the G0/G1 phase.

### 2.4. Pharmacological Inhibition or Genetic Ablation of PANX1 Channels Abrogate Ethidium Bromide (EtBr) Dye Uptake by PANX1 Channels

**Figure 4:**
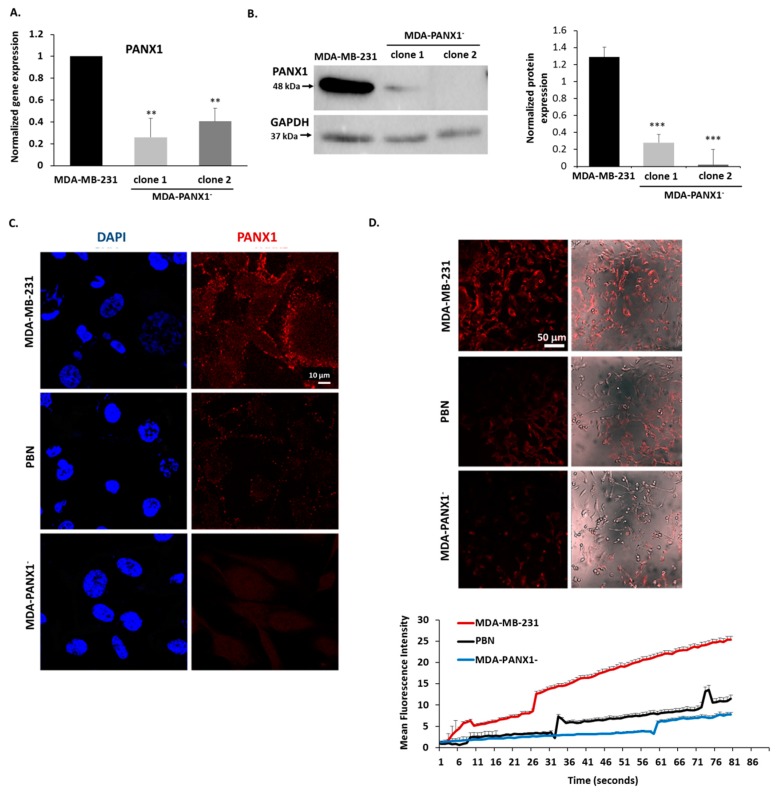
Inhibition of PANX1 channel or genetic ablation of PANX1 gene abrogate ethidium bromide (EtBr) dye uptake by PANX1 channels. (**A**) PANX1 gene was knocked out in MDA-MB-231 cells using CRISPR/Cas9 gene editing. The bar graph shows qRT-PCR results of the mRNA levels of PANX1, normalized to GAPDH, in parental MDA-MB-231 cells and in CRISPR/Cas9 PANX1 knocked-out MDA-MB-231 cells (MDA-PANX1^–^; clone1 and clone 2 cells). (**B**) Representative Western Blot of PANX1 protein levels in parental MDA-MB-231 cells and in MDA-PANX1^–^ cells (MDA-PANX1^–^; clone1 and clone 2 cells). Lower panel is a densitometry quantification of the PANX1 and GAPDH bands using Image Lab software. Values represent the average fold change of PANX1 expression normalized to GAPDH. (**C**) Immunofluorescence images of PANX1 immunostaining in MDA-PANX1^–^ or MDA-MB-231 cells treated with 1 mM PBN for 72 h. Micrographs are representative of three independent experiments. (**D**) Representative fluorescence micrographs of parental untreated MDA-MB-231 cells, MDA-PANX1^–^ or MDA-MB-231 cells treated with 1 mM PBN showing EtBr uptake and mean fluorescence intensity (MFI). Cells, pre-treated with 1 mM PBN for 15 m, were incubated in 25 μM EtBr in low divalent physiological solution. EtBr uptake was monitored by live imaging at 37 °C, for 15 m and images were acquired at 10-second intervals. The MFI of 5 different fields in each micrograph was used to quantify overall fluorescence. Data are displayed as EtBr MFI. Results are representative of three independent experiments. ** *p* < 0.01 and *** *p* < 0.001.

In addition to pharmacological blockade of PANX1 channels, genetic knock-out of PANX1 expression was also performed. CRISPR/Cas9 gene editing system was employed to generate MDA-MB-231 cells with knocked-out PANX1 (MDA-PANX1^–^ cells). MDA-PANX1^–^ cells had diminished levels of PANX1 mRNA, as shown by qRT-PCR (Figure 4A), and protein, as shown by Western blotting (Figure 4B) and immunofluorescence (Figure 4C). 

The probenecid mode of action is thought to be mediated at least in part through the inhibition of PANX1 channels [48]. The dye uptake assay was performed to evaluate the ability of cells to uptake EtBr through PANX1 channels. PBN-treated and control MDA-MB-231 cells were incubated with EtBr. Control cells were able to uptake EtBr, while PBN-treated MDA-MB-231 cells displayed a significant reduction of EtBr uptake as determined by lower EtBr mean fluorescence intensity (MFI; Figure 4D). These data confirm effective inhibition of EtBr dye uptake. The knocked-out PANX1 expression was further confirmed by the lack of EtBr uptake in MDA-PANX1^–^ cells as revealed by dye uptake assay (Figure 4D). The decrease in PANX1 channel activity in MDA-PANX1^–^ cells was similar to that exhibited by PBN-treated MDA-MB-231 cells (Figure 4D). The EtBr dye uptake was also evaluated in the presence of normal divalent ion solution (2 mM CaCl_2_ and 1 mM MgCl_2_), at room temperature and in the presence of 1 mM ATP to activate EtBr uptake by PANX1 [49]. PBN effectively inhibited ATP-induced EtBr dye uptake (Figure S2).

### 2.5. Pharmacological Inhibition or Genetic Ablation of PANX1 Channels Reverse EMT in Breast Cancer Cells

**Figure 5:**
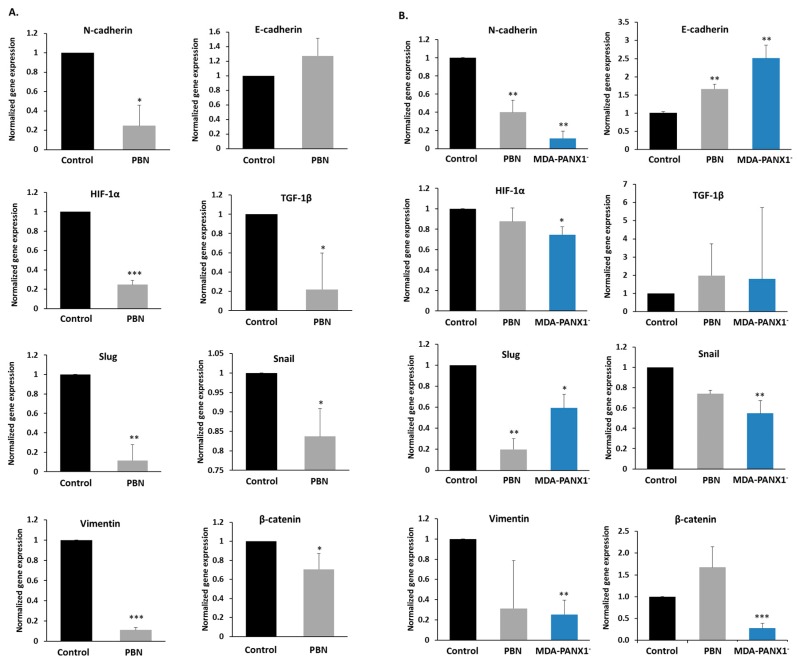
PANX1 channel inhibition or gene ablation downregulate EMT pathway genes in breast cancer cells. mRNA expression levels of EMT genes (N-cadherin, E-cadherin, HIF-1α, TGF-1β, Slug, Snail, Vimentin and β-catenin) in either (**A**) MCF-7 or (**B**) MDA-MB-231 cells treated with 1 mM PBN for 72 h, and MDA-PANX1^–^ cells, quantified by qRT-PCR and normalized to GAPDH, relative to control cells. Bar graphs display results of three independent experiments. * *p* < 0.05, ** *p* < 0.01, and *** *p* < 0.001.

To validate the EMT data obtained in the BRCA TCGA samples in silico, key EMT markers’ expression levels were measured by qRT-PCR in MCF-7 and MDA-MB-231, in the presence or absence of PBN, and in the MDA-PANX1^–^ cells (Figure 5A,B). Upon pharmacological blockade or genetic ablation of PANX1, there was a significant decrease in N-cadherin mRNA levels (*p* < 0.05 for MCF-7 and *p* < 0.01 for MDA-MB-231 cells) paralleled by an increase of E-cadherin mRNA levels (significant only in MDA-MB-231 cells treated with PBN [*p* < 0.01]). MDA-PANX1^–^ cells exhibited similar expression pattern as that of PBN-treated MDA-MB-231 cells, with a significant decrease in N-cadherin mRNA levels (*p* < 0.01) and a significant increase in E-cadherin mRNA levels (*p* < 0.01). Similarly to N-cadherin, vimentin gene expression levels were repressed in both cell lines upon PBN treatment (*p* < 0.001 in MCF-7 cells). Genetic deletion of PANX1 in MDA-MB-231 cells significantly lowered vimentin gene expression levels (*p* < 0.01; Figure 5A,B).

**Figure 6:**
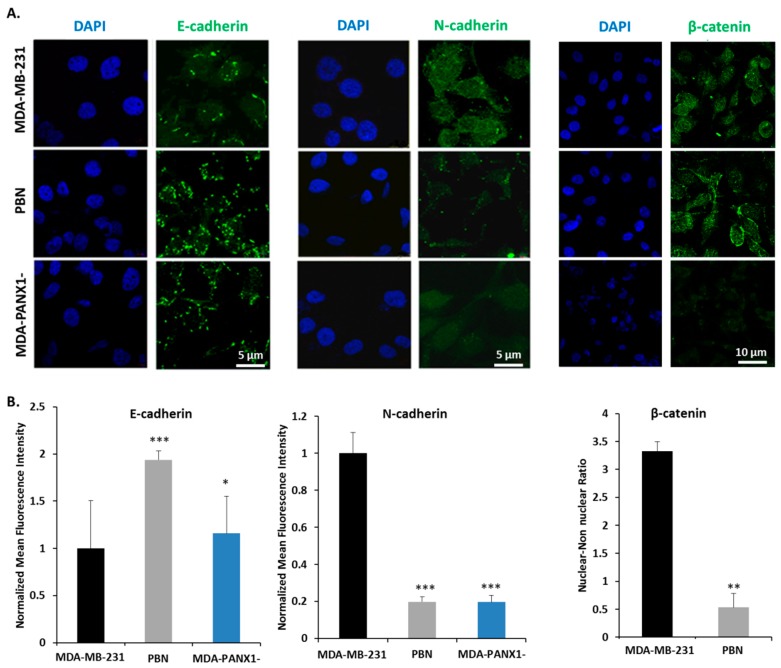
PANX1 downregulation reduces the expression of EMT pathway genes in MDA-MB-231 cells. (**A**) Representative fluorescent micrographs of E-cadherin, N-cadherin, and β-catenin immunostaining in MDA-MB-231 cells, MDA-MB-231 cells treated with 1 mM PBN for 72 h, and MDA-PANX1^–^ cells. (**B**) Quantitative analysis of the data presented in (A). Total mean fluorescence intensity (MFI) was measured in at least five different fields of each of the representative micrographs. Left and middle panels: MFI reflecting amounts of E-cadherin and N-cadherin in PBN-treated and MDA-PANX1^–^ cells, relative to control MDA-MB-231 cells. Right panel: Quantification of nuclear: non-nuclear distribution of β-catenin in MDA-MB-231 cells and in MDA-MB-231 cells treated with 1 mM PBN for 72 h. Micrographs are representative of three independent experiments and bar graphs display average ±SD of MFI. * *p* < 0.05, ** *p* < 0.01, and *** *p* < 0.001.

In addition, mRNA levels of key regulators of EMT that included HIF-1α, Snail, Slug, TGF-1β, and β-catenin were assessed. HIF-1α mRNA levels were significantly decreased in MDA-PANX1^–^ cells (*p* < 0.05, Figure 5B) and in PBN-treated MCF-7 cells (*p* < 0.001, Figure 5A); while PBN exposure did not seem to affect HIF-1α mRNA levels in MDA-MB-231 cells (Figure 5B). Furthermore, a significant decrease in mRNA levels of TGF-1β (*p* < 0.05) and Slug (*p* < 0.01) were exhibited by MCF-7 cells upon PBN treatment (Figure 5A). Probenecid-treated MDA-MB-231 cells had a non-significant decrease in Snail mRNA levels, while MDA-PANX1^–^ cells expressed significantly lower levels of Snail (*p* < 0.01). Notably, β-catenin transcript levels were significantly downregulated in PBN-treated MCF-7 cells (*p* < 0.01), and in MDA-PANX1^–^ (*p* < 0.001). Figure 6 further supports a mesenchymal-epithelial transition (MET) induced by PANX1 downregulation. Immunostaining for E-cadherin and N-cadherin showed enhanced E-cadherin protein levels and reduced N-cadherin protein levels upon PANX1 downregulation, in accordance with the transcriptional data. Furthermore, Figure 6A (right panel) shows that PANX1 downregulation led to more prominent cytoplasmic/membranous distribution and reduced nuclear localization of β-catenin.

Overall, pharmacological or genetic downregulation of PANX1 in breast cancer cells reverses the already existing EMT, i.e., promote a MET state in those cells.

### 2.6. Pharmacological Inhibition or Genetic Ablation of PANX1 Channels Reduce the Metastatic Potential of Breast Cancer Cells

**Figure 7:**
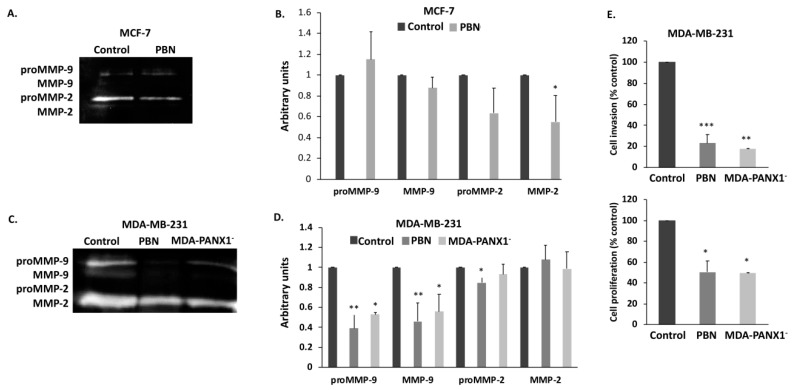
Inhibition of PANX1 channel or genetic ablation of PANX1 gene reduce the invasive and metastatic potential of breast cancer cells. (**A**) Gelatin zymography of 1 mM PBN-treated MCF-7 cells. Proteins were separated on a gelatin-containing polyacrylamide gel in order to assess the activation status and levels of MMP-2 and MMP-9 enzymes. (**B**) Bar graph displays densitometry quantification of zymographs of MCF-7 cells, using Image Lab software. Results are representative of three independent experiments. (**C**,**D**) are the same as in (A,B), respectively, but for MDA-MB-231 treated with 1 mM PBN and for MDA-PANX1^–^ cells. (**E**) RTCA measurements of cell invasion of MDA-MB-231 cells treated with 1 mM PBN for 72 h and of MDA-PANX1^-^ cells. Cell impedance readings were taken every 15 m for a minimum of 18 h. Quantification graphs display normalized cell index values, relative to Control. * *p* < 0.05, ** *p* < 0.01, and *** *p* < 0.001.

Epithelial-to-mesenchymal transition is inherently correlated with the increased metastatic potential of cancer cells. Reversal of EMT upon PBN treatment prompted us to assess the effect of functional inhibition of PANX1 channels on the metastatic potential of breast cancer cells. Given their major role in invasion and metastasis, enzymatic activity of the matrix metalloproteinases MMP-2 and MMP-9 was measured by gelatin zymography in MCF-7 and in MDA-MB-231 cells, in the presence or absence of PBN treatment and in the MDA-PANX1^−^ cells. Data showed a prominent and significant decrease in both proMMP9 (*p* < 0.05 and *p* < 0.01) and MMP-9 (*p* < 0.05 and *p* < 0.01) activity in MDA-MB-231 and in MDA-PANX1^–^ cells (Figure 7C,D). MMP2, on the other hand, showed a slight significant activity decrease in the PBN-treated MDA-MB-231 cell, only (*p* < 0.05). MCF-7 cells, treated with PBN, showed a significant decrease in the activity of MMP2, only (Figure 7A,B, *p* < 0.05).

Decreased metastatic potential of breast cancer cells with diminished PANX1 activity prompted us to assess, using Real-Time Cell Analysis (RTCA), the invasive capacity of MDA-MB-231 cells, upon PBN treatment, and that of MDA-PANX1^-^ cells. MDA-MB-231 cells treated with PBN showed a significant decrease in their invasiveness (80% decrease compared to control, *p* < 0.01) and their proliferation rate (50% decrease compared to control, *p* < 0.01) (Figure 7E). These findings suggest that PANX1 channel permeability blockade or gene deletion decrease the metastatic potential of breast cancer cells MDA-MB-231 cells by decreasing their extracellular matrix penetrating ability and their overall invasive potential.

## 3. Discussion

Connexins mediate direct cell-cell communication [50,51,52] and maintain cell and tissue homeostasis [53,54]. Loss of connexins is a hallmark of cancer progression, including breast cancer [55,56,57]. Pannexins, a vertebrate glycoprotein family [17], share functional and structural features with connexins and also mediate communication between the cell and its microenvironment [20,25]. PANX1, the best characterized member of the pannexin family, plays important roles in tissue development and differentiation [17,58,59,60,61] and is implicated in several human diseases [25]. PANX1 expression has been reported during mouse mammary gland development and in the adult mouse mammary gland [31]; however, its role in breast cancer is still evolving [62]. 

Our results uncovered a role for PANX1 in breast cancer metastasis through EMT regulation. We provide evidence that PANX1 upregulation is correlated with poor prognosis in breast cancer patients and that it is differentially expressed in the different “intrinsic” human breast cancer subtypes. This observation is supported in a previous report by Stewart et al. who correlated PANX1 with negative clinical outcomes leading to poor overall survival and distant metastasis free survival in patients with breast cancer using in silico arrays [31]. Furthermore, Furlow et al. have shown that PANX1 is enriched at metastatic breast cancer foci without any elucidation into the underlying mechanism [30]. Here, we have provided data on pathways and biological processes that are enriched upon PANX1 increased expression. EMT pathway genes, were among the highly enriched gene ontology (GO) pathways following GSEA.

To support our in silico findings we used two human breast cancer cell lines, MCF-7 and MDA-MB-231, these are widely used cell line models to study metastasis [63]. We assessed the effect of PANX1 channel on breast cancer progression via pharmacological inhibition of PANX1 in these cells. Probenecid (PBN), a drug that has been used for gout treatment, is a potent PANX1 inhibitor [48,64,65] and is among several compounds that have been reported to effectively block PANX1 channel activity [66]. Treating MCF-7 and MDA-MB-231 cells with PBN not only decreased PANX1 activity and expression but also significantly reduced the proliferation of these cells by inducing a cell cycle arrest at the G0/G1 transition. Effectively, PBN reduced the number of cycling cells progressing through DNA replication and mitosis. Previously, similar effects on cell viability and PANX1 expression were evidenced in MDA-MB-231 cells [67] and in hippocampal cells from aged rats [68], respectively. In the hippocampal cells, there was a marked reduction in PANX1 protein levels following PBN treatment, similar to the results obtained in this study. The PBN-induced cell cycle arrest can explain its recent use as an effective adjuvant therapy to sensitize breast cancer cells and enhance the efficacy of bisphosphonate chemotherapy [67]. 

Our data show that the inhibition of PANX1 channel activity, using PBN, or the deletion of the PANX1 gene in breast cancer cells, did decrease the mRNA levels of several EMT genes, thus confirming the GSEA in silico data. Importantly, inhibiting PANX1 channels or PANX1 deletion upregulated E-cadherin (an epithelial marker) and down-regulated N-cadherin (a mesenchymal marker). PANX1 upregulation supports an EMT phenotype, while its inhibition or downregulation promotes a mesenchymal to epithelial transition. EMT [34] is crucial in the metastatic process [36] and is associated with tumor cell migration and invasion into the surrounding stroma and dissemination into secondary organs [35,37]. EMT is, in part, mediated through the cell adhesion molecule E-cadherin [38,39]. E-cadherin is crucial in maintaining the epithelial cell phenotype and its downregulation has been correlated with increased invasiveness of breast cancer cells [69]. In addition, the expression of the non-epithelial and mesenchymal-associated molecule N-cadherin contributes to increased invasiveness and motility of breast cancer cells [69,70]. A set of pleiotropically acting transcription factors, including Snail, Slug, and other regulators, orchestrate EMT. Our study revealed a decrease in Snail and Slug mRNA levels in TCGA samples and upon PANX1 downregulation in MCF-7 and in MDA-MB-231 cells. These transcription factors regulate EMT in different types of malignant tumors and their invasiveness. This was confirmed by studies that revealed that EMT transcription factors such as Snail could elicit metastasis when ectopically overexpressed [70,71,72]. We also reported a significant decrease in TGF-1β expression in MCF-7 cells. TGF-1β, known for its anti-proliferative effects and immune evasion by cancer cells [73,74], activates EMT [75,76]. In addition, PANX1 has a role in tumor hypoxia [77]. PANX1 downregulation led to a reduction in the mRNA levels of the hypoxia transcription factor and marker, HIF-1α in breast cancer cells. Hypoxia stabilizes several transcription factors that promote EMT progression and cancer cell metastasis [78,79]. Taken together, we show that ablating the PANX1 gene or inhibiting its activity reverts EMT. This result confirms the GSEA data which demonstrated that PANX1 upregulation correlates with an enhanced EMT state. Similar results have been recently reported in testicular cancer cells, where downregulation of PANX1 or its inhibition downregulated vimentin protein levels and upregulated E-cadherin protein levels, through signal-regulated kinase (ERK) [80]. 

GSEA showed that PANX1 upregulation correlated with the expression of several cell adhesion molecules including β-catenin. Malignant and invasive breast tumors are associated with mutations and over-expression of β-catenin. During EMT, nuclear localization of β-catenin activates target genes, a group of which are EMT genes [41,44]. The β-catenin/TCF/LEF transcription complex directly upregulates genes associated with EMT, such as EMT transcription factors Snail1 and Zeb1 [41,44,45]. Snail1 and Zeb1 act as transcription repressors of E-cadherin, leading to down-regulation of E-cadherin expression during EMT [46,47]. Of these established EMT markers, our study revealed that Snail and Slug transcription factors highly correlate with PANX1 expression. PANX1 could possibly act indirectly by regulating genes such as β-catenin which then regulate EMT. On the other hand, a direct role for PANX1 as a transcription regulator of EMT in the nucleus is plausible. This direct role for PANX1 is not far-fetched since such a role has been reported for Connexin 43 [81], a protein that shares structural and functional similarities with PANX1. However, this mechanism requires further scrutiny. 

Interestingly, the decreased expression of EMT key markers, upon PANX1 inhibition or genetic deletion, was also correlated with decreased matrix metalloproteinases (MMPs) activity in MCF-7 and MDA-MB-231 cells, and decreased MDA-MB-231 cells invasion potential. MMPs play a vital role in the cancer microenvironment and are markers of breast cancer patients’ poor prognosis [82]. Reduced MMP activity and invasiveness are common characteristics of less metastatic forms of cancer [82]. Overall, a pathway of PANX1 action could be envisaged to explain why PANX1 overexpression correlates with poor prognosis in breast cancer patients. In this pathway, upregulated PANX1 enhances breast cancer cells EMT phenotype, the enhanced EMT phenotype enhances breast cancer cells metastatic potential and invasiveness, for example by increasing the levels or activity of MMPs and other metastatic genes. This scenario is supported by data from recent studies on testicular cancer cells, where PANX1 inhibition downregulates EMT genes, upregulates E-cadherin, downregulates MMP-9, and decreases cell invasion [80]. Freeman et al. showed that decreasing PANX1 protein levels, in melanoma cells, using shRNA or blocking PANX1 channel using PBN or carbenoxolone decreased cell growth, migration, and invasiveness, probably through the Wnt/β-catenin pathway [83]. 

Together, our findings identify a role for PANX1 in breast cancer progression and that PANX1 mediates this tumor-promoting role by modification of the EMT pathway.

## 4. Materials and Methods

### 4.1. Cell Culture

Human luminal breast ductal adenocarcinoma epithelial MCF-7 cell line (ER+ PR+ HER2^–^) [84] and triple negative adenocarcinoma epithelial MDA-MB-231 cell line, characterized by its highly metastatic and invasive properties [84,85], were grown in RPMI-1640 medium (Sigma, St. Louis, MO, USA), supplemented with 10% fetal bovine serum (FBS, Sigma) penicillin/streptomycin (P/S, 100 units of potassium penicillin and 100 μg of streptomycin sulfate per 1 mL of medium, Sigma). 

Probenecid (PBN, Santa Cruz Biotechnology, Dallas, TX, USA), also known as p-(dipropylsulfamoyl) benzoic acid, is a PANX1 channel inhibitor [48]. PBN was dissolved in DMSO and DMSO-treated cells were used as vehicle control.

### 4.2. CRISPR/Cas9 Mediated Targeting of Human PANX1 

To knock out PANX1 gene from MDA-MB-231 cells, gRNA oligo pairs targeting exon 2 of human PANX1 were designed with high specificity and low off-targets using the CRISPOR software (www.crispor.tefor.net) and inserted into the plasmid pX330-puro-Rosa26-H3F3B, (Addgene plasmid # 73131; a kind gift from Dr. Agnel Sfeir [86]). MDA-MB-231 cells at 70–80% confluence were transfected with the vector containing the PANX1 gRNA, using Lipofectamine 2000 (Invitrogen, Carlsbad, CA, USA). Limiting dilution cloning was performed by trypsinizing the cells and seeding them at a density of 0.8 cell per well of a 96-well plate. After 10 days of selection in media containing 1 µg/mL puromycin, growing colonies were isolated and transferred to larger culture plates for further expansion. The selected and expanded colonies were further grown and harvested for RNA and protein analysis. Knock-out of PANX1 expression was confirmed by qRT-PCR, Western blotting, and immunofluorescence. MDA-MB-231 cells with knocked-out PANX1 are designated as MDA-PANX1^–^ cells. Mock-transfected MDA-MB-231 cells were generated by transfection of an empty pX330-puro-Rosa26-H3F3B plasmid without any targeting gRNAs into MDA-MB-231 cells followed by selection in puromycin as described above and these cells were used as a control to MDA-PANX1^-^ cells.

### 4.3. In Silico Gene Expression Analysis

#### 4.3.1. Transcriptomic Datasets

BRCA RNAseq data were acquired from The Cancer Genome Atlas (TCGA) database [87]. Gene expression values were represented as Fragment Per Kilobase Million mapped reads (FPKM). Prior to analysis, FPKM values were transformed to log2 scale after the addition of one (log2(x+1). Clinical annotations were obtained in R environment using cbioportal’s cgdsr package [88,89]. The dataset contained 1071 BRCA and 113 normal breast tissue samples. Among the normal tissues, a total of 109 samples had paired BRCA tissues while 4 remained unmatched and were, thereby, excluded. ERα, PR and HER2 status was determined by immunohistochemistry in the initial TCGA publication [87]. Normalized microarray data from the Molecular Taxonomy of Breast Cancer International Consortium (METABRIC) [90] and corresponding clinical annotations were downloaded from cbioportal using the cgdsr package.

#### 4.3.2. Gene Set Enrichment Analysis (GSEA)

GSEA was performed to uncover Kyoto Encyclopedia of Genes and Genomes (KEGG) and Gene Ontology (GO) biological processes that are positively correlated with PANX1 expression. KEGG and GO biological process gene sets were downloaded from the Molecular Signatures Database (MSigDB) (http://www.broadinstitute.org/gsea/). Analysis was done in the desktop GSEA software developed by the Broad Institute [91,92]. Genes were ranked based on their Pearson correlation with PANX1 expression level. Enrichment analysis was performed using 1000 phenotype permutations, and gene sets with nominal *P* value < 0.05 and false discovery rate (FDR) < 0.25 were considered significant. 

#### 4.3.3. Proteomic Analysis

Isobaric Tags for Relative and Absolute Quantification (iTRAQ) proteomics data of 105 TCGA BRCA samples were collected from the Clinical Proteomic Tumor Analysis Consortium (CPTAC) data portal [93]. Analyzed samples covered 4 BRCA subtypes: Luminal A, Luminal B, Basal-like, and HER2-enriched. Protein expression was calculated as log2 Ratio to a pool of internal reference comprising a mixture of 40 samples with equal representation of the 4 breast subtypes. Among the 105 available samples, only 72 samples possessed PANX1 protein expression data.

### 4.4. Survival Analysis

Overall survival (OS) was defined as the duration from the time of diagnosis (in months) until death. Patients of TCGA or METABRIC data sets were segregated into High, Intermediate or Low PANX1 expression score groups based on the 25th and 75th percentiles of PANX1 expression. PANX1 High, Intermediate, and Low expression score groups were plotted using Kaplan–Meier survival curves. Kaplan–Meier plots were used to compare OS of High/Intermediate versus Low PANX1 expression groups.

### 4.5. Patients and Specimens

Breast carcinoma samples were retrieved from the pathology departments at the American University of Beirut Medical Center and Hammoud Hospital University Medical Center, after obtaining approval from the ethics committee (IRB reference number: PALM. FB. 01). A total of 37 patients with matched age (over 50 years old) and no treatment history were classified into 3 groups. Group I TNBC (11 specimens of triple negative breast cancer [TNBC]), group II HER2^+^ (11 specimens of estrogen receptor [ER]^−^/progesterone receptor [PR]^–^/human epidermal growth factor receptor 2 [HER2]^+^ breast cancer), and group III HER2^–^ (15 specimens of ER^+^ PR^+^ HER2^–^ breast cancer). All groups were evaluated for histologic subtype, grade, central fibrosis, and tumor necrosis. A representative formalin-fixed paraffin-embedded (FFPE) tissue block from each case was obtained for molecular analyses. Negative controls were obtained from breast tissue of patients who underwent reduction mammoplasty [94]. The protocol of this study was approved by the American University of Beirut under the permission of the pathology departments at the American University of Beirut Medical Center and Hammoud Hospital University Medical Center (IRB PALM. FB. 01). All patients provided written informed consent to the surgical procedures and gave permission for the use of resected tissue specimens.

### 4.6. Cell Viability Assay

MCF-7 and MDA-MB-231 cells were seeded in 24-well cell culture plates at a density of 1.5 × 10^4^ cells per well. At 30–40% confluence, cells were treated with different PBN concentrations (0.1, 0.3, 0.5, and 1 mM). Cells were harvested by trypsinization at 24, 48, and 72 h following treatment, then centrifuged at 200× *g* for 5 m. Obtained cell pellets were reconstituted in culture media and Trypan Blue (Sigma) dye exclusion assay was performed. Cells were counted using a hemocytometer.

### 4.7. Cell Cycle Analysis

MCF-7 and MDA-MB-231 cells were seeded in 6-well plates at a density of 8 × 10^4^ cells per well and incubated for 24 h prior to treatment with 0.5 mM or 1 mM PBN. Cells were harvested at 24, 48, and 72 h following treatment, washed twice with phosphate-buffered saline (PBS), centrifuged at 200× *g* for 5 m at 4 °C, re-suspended in 1 mL of old PBS and fixed in 4 mL of cold absolute ethanol. Prior to flow cytometry analysis, cells were pelleted and treated with 100 µL of 200 µg/mL DNase-free RNase A for an hour at room temperature, and then incubated with 20 μg/mL propidium iodide ([PI]; Sigma) for 15 m in the dark. Cell cycle analysis was performed using a Guava Easy Cyte 8™ flow cytometer operated by Guava InCyte™ software (EMD Millipore, Darmstadt, Germany).

### 4.8. Gene Expression Analysis by Quantitative Real-Time Polymerase Chain Reaction (qRT-PCR)

Gene expression levels of PANX1 were determined in archived breast cancer tissues. mRNA levels of PANX1 along with EMT markers were also determined in MCF-7, MDA-MB-231 and MDA-PANX1^–^ cells by qRT-PCR. Real time-PCR primers are listed in Table 1. Briefly, extraction of total RNA from cells was performed using RNeasy^®^ Plus Mini Kit (QIAGEN, Hilden, Germany) while extraction of RNA from mouse or human tissues was performed using TRIzol^®^ Reagent (Invitrogen), following the manufacturer’s protocols and as previously described [95]. One µg of total RNA was reverse-transcribed to a single stranded complementary DNA (cDNA) using Revert Aid first strand cDNA synthesis kit (Thermo, Waltham, MA, USA). qRT-PCR was performed using iQ SYBR Green Supermix (Bio-Rad, Hercules, CA, USA) in a CFX96™ Real-Time PCR Detection System (Bio-Rad). PCR amplification steps were as follows—95 °C for 10 s annealing temperature of the target gene for 30 s and then 72 °C for 30 s. The fluorescence threshold cycle value was obtained for each gene. ΔΔCq method was used to calculate the relative fold change in gene expression after normalization to the housekeeping gene, GAPDH.

### 4.9. Protein Expression Analysis by Western Blotting

Proteins from cultured cells were extracted using a lysis buffer (126 mM Tris/HCl, 20% glycerol (v/v), 40 mg/mL of sodium dodecyl sulfate [SDS]) containing protease and phosphatase inhibitors (Roche, Penzberg, Germany). Protein concentrations were determined using DC Protein Assay II kit (Bio-Rad). Total protein lysates (100 µg) were resolved by SDS-PAGE and then transferred to PVDF membranes (Bio-Rad). The PVDF membranes were blocked with 5% skimmed milk for 1 hour before incubation with primary antibodies at a concentration of 1 µg/mL: anti-PANX1 (cat #710184, Invitrogen, Carlsbad, CA, USA) and anti-GAPDH (cat #G8795, Sigma). The membranes were then washed in PBS and incubated with appropriate horseradish peroxidase-conjugated IgG secondary antibody (Santa Cruz Biotechnology). Protein bands were visualized using chemiluminescence, and their intensity was quantified by densitometry and normalized to GAPDH using the Image J software (https://imagej.nih.gov/ij/).

### 4.10. Invasion and Proliferation Assays

Real-Time Cell Analysis (RTCA) was used to study invasion and proliferation of MDA-MB-231 and MDA-PANX1^–^ cells. Cells were seeded on a cellular invasion plate (CIM-plate 16) with micro-electronic sensors on the underside of an 8 µm microporous polyethylene terephthalate membrane [18,96,97,98] of a Boyden-like upper chamber, and real-time evaluation of cell impedance was performed using xCELLigence RTCA [A2] DP instrument (Roche). For invasion assays, 30 µL of growth factor-reduced Matrigel^TM^ (BD Biosciences, San Jose, CA, USA) diluted in serum-free medium at a ratio of 1:20 were used to coat the upper surface of the membrane, followed by incubation at 37 °C in 5% CO_2_ for 4 h in a humidified incubator and then washed with PBS. A volume of 160 µL of RPMI-1640 containing 10% FBS was added to the lower chamber of each well and 30 µL to the upper chamber. The plate was incubated for 1 hour at 37 °C before cell seeding. Cells were seeded in the upper chamber at a density of 2 × 10^4^ cells per 100 µL of serum-free media. MDA-MB-231 cells were allowed to adhere before PBN (1 mM) treatment was applied. For cell proliferation assay, cells were seeded in an E-plate as described above, at a density of 1 × 10^4^ cells with an additional 150 µL of media containing 10% FBS. Both invasion and proliferation were monitored by recording cell impedance every 15 m for a minimum of 18 h.

### 4.11. Gelatin Zymography

Cell culture conditioned media were collected 48 h following incubation of MCF-7, PBN-treated MCF-7, MDA-MB-231, PBN-treated MDA-MB-231 and MDA-PANX1^–^ in serum-free media, and the enzymatic activity of metalloproteinase (MMP)-2 and MMP-9 was assessed by gelatin zymography. Concentrated protein supernatants (70 µg) were run on an SDS-PAGE gel, containing gelatin as a substrate. Gels were then stained with Coomassie^TM^ brilliant blue R-250 (Bio-Rad) for one hour at room temperature and de-stained with a solution of methanol, acetic acid, and water. Band staining intensity was determined by densitometry, using Image J software.

### 4.12. Dye Uptake Assay

MDA-MB-231 and MDA-PANX1^–^ cells were seeded onto glass-bottom dishes (confocal dishes, MatTek Corporation, Ashland, MA, USA), at a seeding density of 1.2 × 10^4^ cells per dish. At 60–70% confluence, cells were washed twice with 200 µL of low divalent ion extracellular solution (145 mM NaCl, 5 mM KCl, 13 mM glucose, 0.2 mM CaCl_2_, 10 mM Hepes, pH 7.3). MDA-MB-231 cells were then treated with 1 mM PBN for 15 m at 37 °C, before applying low divalent ion extracellular solution containing 25 µM ethidium bromide ([EtBr]; MP Biomedicals, Germany) [99]. Measurement of EtBr uptake by the cells was performed by recording EtBr fluorescence by live imaging for 15 m at 37 °C using a Laser Scanning Confocal Microscope (LSM710, Carl Zeiss, Oberkochen, Germany). The assay was also performed in normal divalent solution (145 mM NaCl, 5 mM KCl, 2 mM CaCl_2_, 1 mM MgCl_2_, 13 mM glucose, 10 mM HEPES, pH 7.3) and at room temperature, with the addition of 1 mM ATP to stimulate PANX1 channel opening, as previously documented [49]. Results from this assay are displayed in Figure S2. 

### 4.13. Protein Localization by Immunofluorescence

MDA-MB-231 and MDA-PANX1^–^ cells were grown on glass coverslips, fixed with 4% paraformaldehyde (PFA) for 15 m and permeabilized with 0.3% Triton™ X-100 for 10 m. Cells were washed 3 times with PBS and blocked with 1% bovine serum albumin (BSA) for 1 hour at room temperature in a humidified chamber. Cells were incubated overnight at 4 °C with 2 µg/mL of PANX1 antibody (cat #710184, Invitrogen, Carlsbad, CA, USA), E-cadherin antibody (cat# sc-8426, Santa Cruz), N-cadherin antibody (cat# 33–3900, Invitrogen, Carlsbad, CA, USA) or β-catenin antibody (cat# 9582S, Cell Signaling, Danvers, MA, USA) in 0.1% BSA blocking solution. This incubation was followed by washing with PBS and incubation with 1 μg/mL IgG-Texas Red-conjugated secondary antibodies (cat #: 2015320, Invitrogen, Carlsbad, CA, USA) or FITC-conjugated secondary antibodies (cat# F2765, ThermoFisher-Molecular Probes, Eugene, OR, USA) for 1 hour, in the dark at room temperature according to manufacturer protocol. Nuclei were counterstained with 1 μg/mL 4′, 6-diamidino-2-phenylindole ([DAPI]; ThermoFisher-Molecular Probes) for 10 m, followed by 3 washes with PBS and coverslips were then mounted on glass slides using Prolong Antifade reagent (Invitrogen, Carlsbad, CA, USA) and observed under the LSM710 fluorescent microscope. The mean fluorescence intensity (MFI) of 5 different fields in each micrograph of 3 independent experiments was used to quantify overall fluorescence. Average MFI was then displayed in bar graphs.

### 4.14. Statistical Analysis

For survival analyses, the Log-rank test was used to estimate significance and hazard ratio (HR). Univariate Cox regressions were performed using dichotomic PANX1 score as variable. Prognosis significance was estimated using Wald’s *p* value. Analyses were performed in R environment using survival and survminer packages (https://CRAN.R-project.org/package = survival; https://CRAN.R-project.org/package = survminer).

Histograms, boxplots, and correlation plots were generated in R using ggpubr (https://CRAN.R-project.org/package = ggpubr). Pearson’s method was used for gene correlation analysis in R as implemented in the corrplot package (https://github.com/taiyun/corrplot). Adjustment for multiple comparisons was done using the Benjamini and Hochberg method [100]. 

Microsoft Excel and GraphPad Prism software were also used to perform statistical analysis. Results are expressed as individual data or as mean ± standard deviation. Student’s *t*-test was used to compare various groups. Statistical significance was determined by unpaired Student’s *t*-test. 

Differences between groups were assessed by one-way analysis of variance (ANOVA). 

*p* values were determined and values of *p*  <  0.05, *p* < 0.01, *p* < 0.001 (*, **, *** respectively) were considered significant.

## 5. Conclusions

This study supports a role for PANX1 in cancer progression and metastasis, where its expression is upregulated in all breast cancer subtypes and upregulated PANX1 levels in breast cancer tissues are correlated with poor clinical outcomes. Furthermore, this study uncovers a role for PANX1 in EMT regulation in breast cancer. Overall, the ablating PANX1 function, pharmacologically or genetically, may constitute a novel multi-pronged therapeutic target for metastatic breast cancer by reverting EMT and decreasing the metastatic ability and invasiveness of breast cancer cells.

## Figures and Tables

**Table 1:** List of human primers for qRT-PCR.

Genes	Primer Sequence	Annealing Temperature (°C)
**PANX1**	F: AGACGAGTTTGTGTGCAGCATC R: CAAAAGTGGGGAGGATTCGTAC	56
**E-cadherin**	F: CAGAAAGTTTTCCACCAAAG R: AAATGTGSGCAATTCTGCTT	58
**N-cadherin**	F: GGTGGAGGAGAAGAAGACCAG R: GGCATCAGGCTCCACAGT	58
**HIF-1α**	F: AGCCAGATCTCGGCGAAGT R: CAGAGGCCTTATCAAGATGCG	58
**TGF-1β**	F: CTAATGGTGGAAACCCACAACG R: TATCGCCAGGAATTGTTGCTG	57
**Slug**	F: GAGCATTTGCAGACAGGTCA R: ACAGCAGCCAGATTCCTCAT	57
**Snail**	F: CTTCCAGCAGCCCTACGAC R: CGGTGGGGTTGAGGATCT	58
**Vimentin**	F: AGGTGGACCAGCTAACCAAC R: TCTCCTCCTGCAATTTCTCC	56
**β-catenin**	F: AGGGATTTTCTCAGTCCTTC R: CATGCCCTCATCTAATGTCT	54
**GAPDH**	F: TGGTGCTCAGTGTAGCCCAG R: GGACCTGACCTGCCGTCTAG	58

F: Forward primer sequence; GAPDH: Glyceraldehyde 3-phosphate dehydrogenase; HIF-1α: Hypoxia-inducible factor 1-alpha; PANX1: Pannexin 1; R: Reverse primer sequence; TGF-1β: Transforming growth factor beta 1.

## Supplementary Materials

The following are available online at https://www.mdpi.com/2072-6694/11/12/1967/s1, Figure S1: PANX1 expression does not correlate with age. Figure S2: PBN attenuates ATP-induced EtBr dye uptake by PANX1 channels; Figure S3: Western blot images captured on the Chemidoc MP system; Table S1: Intensity ratios obtained by densitometry of PANX1 bands, normalized to densitometry of GAPDH bands, using Image Lab software.
