# Supplementary material for: Pannexin1 Is Associated with Enhanced Epithelial-To-Mesenchymal Transition in Human Patient Breast Cancer Tissues and in Breast Cancer Cell Lines
Source: Cancers (Basel). 2019 Dec 7;11(12):1967. doi: 10.3390/cancers11121967 (PMC6966616; doi:10.3390/cancers11121967)
Supplement: Supplementary file 1 [file cancers-11-01967-s001.pdf]

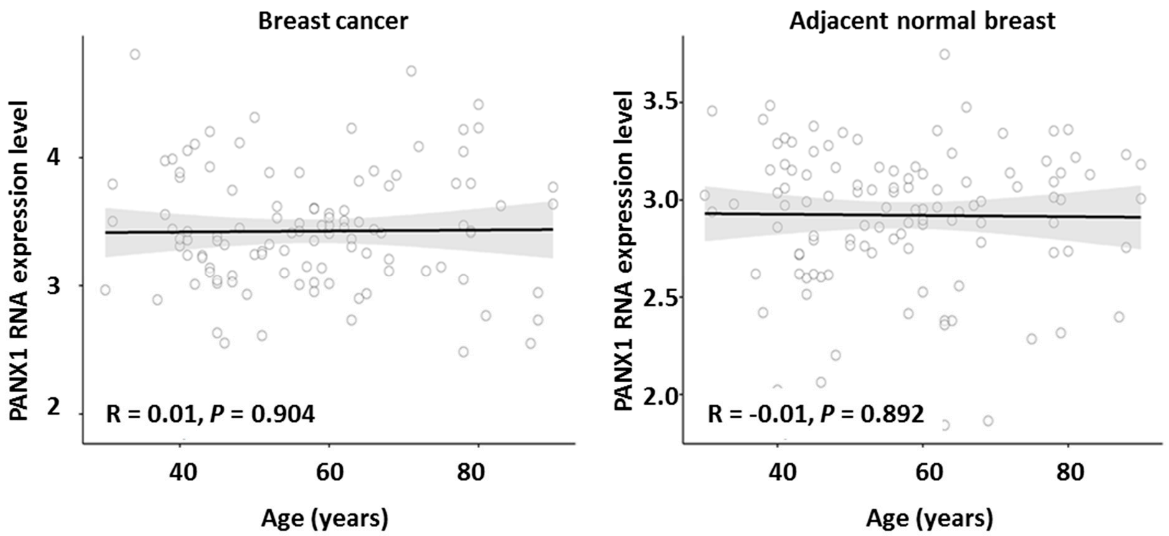

Figure S1: **PANX1 expression does not correlate with age.** Scatter plots representing the analysis of PANX1 mRNA expression correlation with age in breast cancer tissues (P = 0.904) and in adjacent non-cancerous breast tissues (P = 0.892).

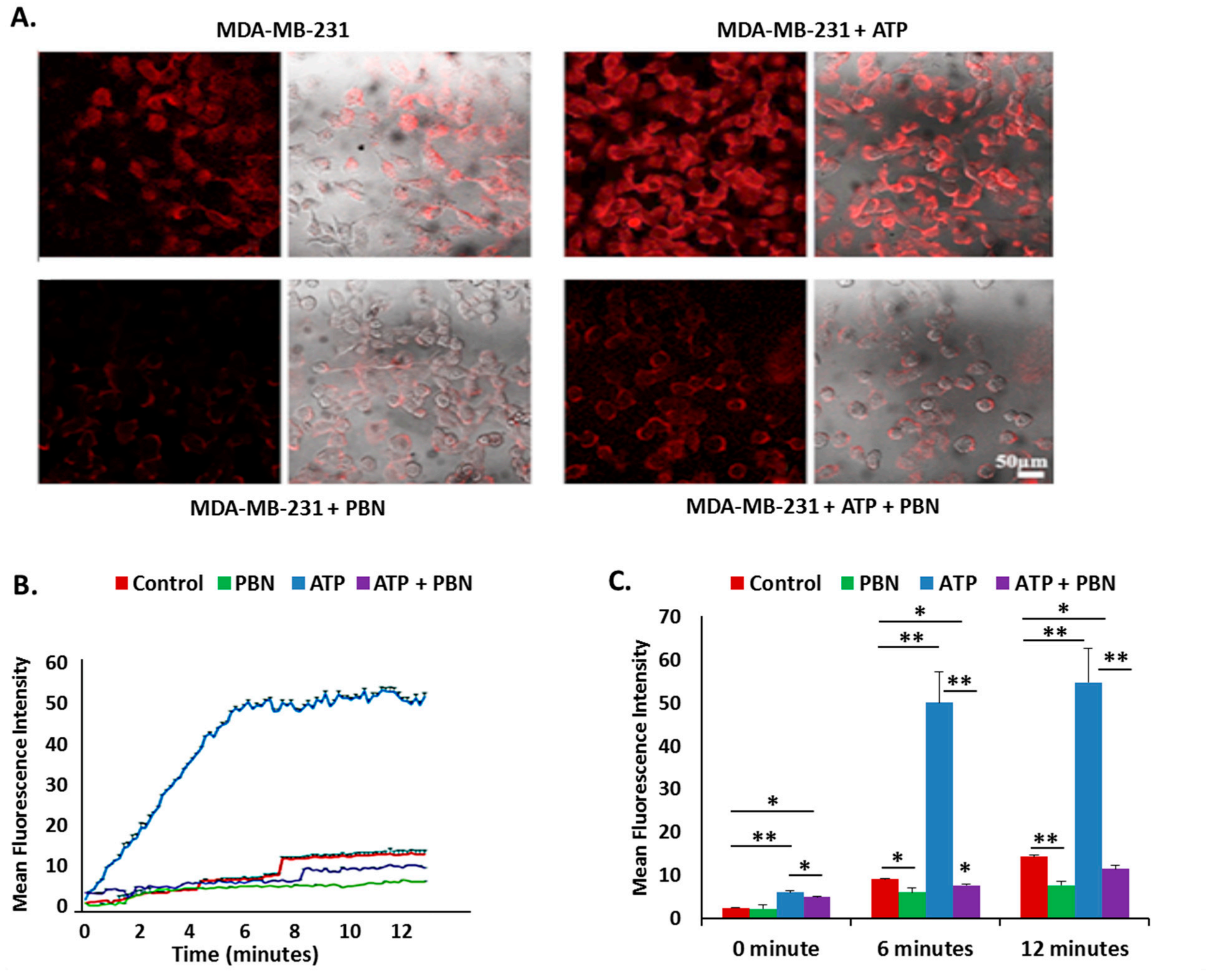

Figure S2: **PBN attenuates ATP-induced EtBr dye uptake by PANX1 channels.** (A) Representative fluorescence micrographs of EtBr uptake. EtBr dye uptake was induced in untreated MDA-MB-231 cells and in MDA-MB-231 cells pre-treated with 1 mM PBN, by the addition of 1 mM ATP in normal divalent physiological solution (2 mM Ca^{2+} and 1 mM Mg^{2+}) and at room temperature. (B) Kinetic traces of the conditions specified in (A); Dye uptake was recorded by live imaging and images were acquired at 10-second intervals. The mean fluorescence intensity (MFI) of 5 different fields in each micrograph was used to quantify overall fluorescence. Data are displayed as EtBr MFI. (C) Summary of dye uptake assays in (B); Bar charts represent MFI calculated at 0, 6 and 12-minute timepoints for the different conditions. Results are representative of 2 independent experiments. $*P < 0.05$, $**P < 0.01$, and $***P < 0.001$.

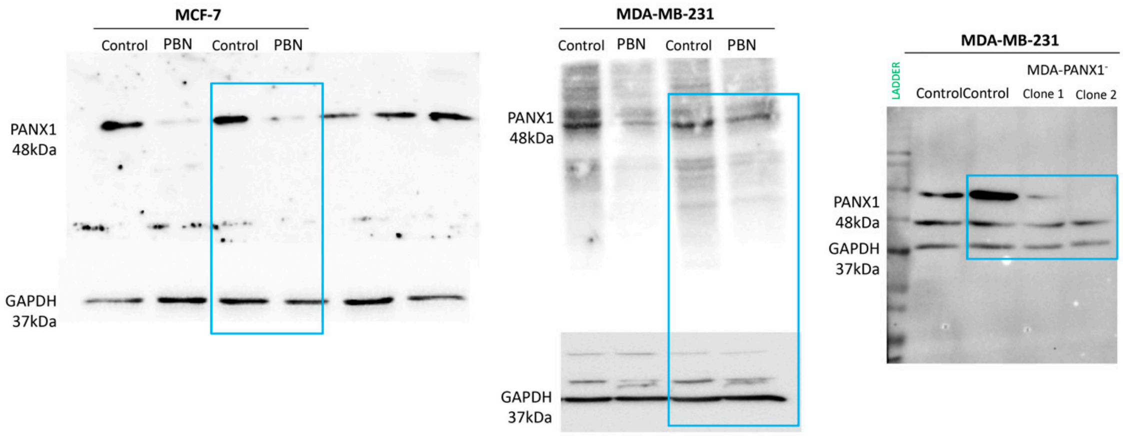

Figure S3: Original western blot images captured using the BioRad Chemidoc MP system.

Table S1: Intensity ratios of the densitometry analysis of PANX1 and GAPDH western blot bands

		Run 1	Run 2	Run 3	Average	Standard Deviation	*p* value
**MCF-7**	**Control**	0.778	0.607	0.626	0.670	0.093	
	**PBN**	0.137	0.133	0.133	0.134	0.002	$p < 0.005$
**MDA-MB-231**	**Control**	0.859	1.044	0.631	0.845	0.207	
	**PBN**	0.520	0.384	0.510	0.471	0.075	$p < 0.05$
	**Control**	1.427	1.213	1.450	1.363	0.131	
**MDA-PANX1$^-$**	**Clone 1**	0.670	0.404	0.382	0.485	0.160	$p < 0.005$
	**Clone 2**	0.838	0.609	0.755	0.734	0.116	$p < 0.005$

PBN: Probenecid
P values were calculated according to Student's *t*-test.
